# Risk factors and obstetric complications of large for gestational age births with adjustments for community effects: results from a new cohort study

**DOI:** 10.1186/1471-2458-10-460

**Published:** 2010-08-06

**Authors:** Shu-Kay Ng, Adriana Olog, Anneliese B Spinks, Cate M Cameron, Judy Searle, Rod J McClure

**Affiliations:** 1 School of Medicine, Griffith University (Logan Campus), Meadowbrook, QLD 4131, Australia; 2 Department of Obstetrics and Gynaecology, Gold Coast Hospital, Australia; 3 Commonwealth Scientific & Industrial Research Organisation, Australia; 4 Health Workforce Division, Department of Health & Ageing, Australia; 5 Monash University Accident Research Centre, Monash University, Victoria, VIC 3800, Australia

## Abstract

**Background:**

High birth weight has serious adverse impacts on chronic health conditions and development in children. This study identifies the social determinants and obstetric complications of high birth weight adjusted for gestational age and baby gender.

**Methods:**

Pregnant women were recruited from three maternity hospitals in South-East Queensland in Australia during antenatal clinic visits. A questionnaire was completed by each participant to elicit information on eco-epidemiological exposures. Perinatal information was extracted from hospital birth records. A hierarchical mixture regression model was used in the analysis to account for the heterogeneity of birth weights and identify risk factors and obstetric complications of births that were large for gestational age. A generalized linear mixed model was used to adjust for (random) "community" effects.

**Results:**

Pre-pregnancy obesity (adjusted OR = 2.73, 95% CI = 1.49-5.01), previous pregnancy (adjusted OR = 2.03, 95% CI = 1.08-3.81), and married mothers (adjusted OR = 1.85, 95% CI = 1.00-3.42) were significantly associated with large for gestational age babies. Subsequent complications included the increased need for delivery by caesarean sections or instrumental procedures (adjusted OR = 1.98, 95% CI = 1.10-3.55), resuscitation (adjusted OR = 2.52, 95% CI = 1.33-4.79), and transfer to intensive/special care nursery (adjusted OR = 3.76, 95% CI = 1.89-7.49). Communities associated with a higher proportion of large for gestational age births were identified.

**Conclusions:**

Pre pregnancy obesity is the principal modifiable risk factor for large for gestational age births. Large for gestational age is an important risk factor for the subsequent obstetric complications. The findings improve the evidence-base on which to base preventive interventions to reduce the impact of high birth weight on maternal and child health.

## Background

Increased numbers of high birth weight infants (>4000 g) and large for gestational age (birth weight above the 90^th ^percentile for gestational age) have been reported in North America and Europe [1-3]. Macrosomia, defined by the American College of Obstetricians and Gynecologists, as birth-weight >4000 or >4500 g irrespective of gestational age is associated in the literature with numerous perinatal and maternal complications. Macrosomic infants are at an elevated risk of shoulder dystocia and associated brachial plexus injury, perinatal asphyxia, meconium aspiration, hypoglycaemia and fetal death [4,5]. Associated maternal complications include prolonged labour, labour augmentation with oxytocins, caesarean delivery, prolonged hospital stay and higher mortality from coronary heart disease for the mother [6-8].

Children born large for gestational age are prone to induce neonatal complications [9] and develop insulin resistance [10], obesity, diabetes and early cardiovascular disease later in life [11-13]. High birth weight has also been associated with increased future risk of cancer such as leukemia, breast, prostate and colon cancer [14,15]. Large for gestational age births have increased from 9.2% to 10.8% in male infants and from 9.1% to 11% in female infants from 1990 to 2005 [16].

High birth weight is also associated with subsequent childhood and adult obesity [12,13,17]. The long-term chronic disease consequences of childhood overweight or obesity are of serious public health concern [18]. The proportion of overweight or obese children in Australia has been increasing at an accelerating rate since the 1980's, with obesity increasing 2-4 times, and being overweight increasing by 60-70% [19,20]. The reported prevalence of overweight or obesity in an Australian population is 34% [21]. The increased prevalence has lead to obesity being recognized as a national health priority risk factor in Australia [22].

The use of risk factor information to identify mothers at risk of having large for gestational age births is an important clinical tool as the accuracy of weight estimation in the third trimester, whether by clinical estimation or ultrasound is poor [23]. Although some causes for large for gestational age births (such as maternal obesity and diabetes) are known, some causes of large for gestational age births are of unknown origin [9,10]. Previously identified risk factors in the literature associated with increased birth weight are maternal obesity, multiparity, advanced maternal age, ethnicity, excessive weight gain, marital status, smoking, prolonged labour [24]. However, the extent to which each of these factors influence birth weight is unclear. There remains substantial variation in the literature regarding the strength of association between each of the identified risk characteristics and macrosomia.

In this paper, we aim to refine knowledge of the social determinants of large for gestational age births and assess the subsequent obstetric complications adjusted for gestational age and baby gender, on the basis of the first three phases of a new 'Environments for Healthy Living' birth cohort study using a hierarchical mixture regression model. The identification of modifiable risk factors of large for gestational age births may contribute to the development of public health interventions to reduce the escalating burden resulting from high birth weight in Australia.

## Methods

### Study design

The birth cohort study 'Environments for Healthy Living' was launched in November 2006 to quantify the relationship between social, environmental and behavioural factors and the health and development of children in South East Queensland, Australia. The study area contains an estimated population of over 1,300,000 people or approximately 4% of Queensland's population. The study region is markedly heterogeneous with respect to age and socioeconomic distribution [25].

Eligible participants were infants of mothers who gave birth at one of three maternity hospitals (Logan, Gold Coast and The Tweed Hospitals) in South East Queensland between November 2006 and August 2008. All women waiting for third trimester antenatal clinic appointments at each of the locations were approached by research trained midwives, provided with a detailed explanation of the study aims and invited to participate in the study. Written informed consent was obtained for release of hospital perinatal data related to the birth of their child, completion of a participant baseline survey and for individual follow-up. Ethics approval for participant recruitment and follow-up of the 'Environments for Healthy Living' birth cohort was obtained from the Griffith University Ethics Committee (Reference number: MED/16/06/HREC) and the Human Research Ethics review Committees of the three participating public maternity hospitals in the study area (reference numbers: 200652, 2006/096, and 358N).

A questionnaire was completed by each participant to elicit information on demographics, socioeconomic status, family structure and relationship, neighbourhood and community connectedness, maternal smoking and drinking behaviour, and the usage of supplements and recreational substances during pregnancy. Perinatal information was extracted from hospital birth records. The Environments for Healthy Living study is based on an ecological model of causation, which attempts to investigate effective social and economic approaches for improving the health of disadvantaged populations and contributing to overall health and wellbeing of populations. A wide variety of health-related exposures and outcomes are measured at baseline and during subsequent follow-up period. The present research extracts variables collected at baseline under the following eco-epidemiological headings: (1) Demographics; (2) Socio-economics; (3) Psychological and behavioural; (4) Social network and neighbourhood; (5) Birth procedures; and (6) Neonatal. The first four eco-epidemiological categories are potential risk factors for large for gestational age births. Variables in the last two categories are adopted to assess potential obstetric complications of delivering large for gestational age babies. All these variables are included in the subsequent analyses.

### Analytic strategy

Identification of risk factors for large for gestational age births is usually undertaken using a logistic regression approach with dichotomous outcomes of large for gestational age defined by birth weight percentile for gestational age [26]. The large for gestational age variable is usually defined on the basis of local growth charts specific for gender and gestational age [27] and thus the definition of large for gestational age infants is subjective to the reference adopted. As mean birth weight has continuously increased in the United States, Canada, Europe, and Asia [1,2], an up-to-date local reference may not be always available. The adoption of an inappropriate reference can result in misleading inference, with the consequent possibility of invalid findings. Moreover, the logistic regression approach is not able to account for heterogeneity as well as variability of birth weights simultaneously. In this paper, a hierarchical mixture regression model [28] has been adopted to simultaneously account for the heterogeneity of birth weights (via mixture modelling) and adjust for risk factors and complication variables (via logistic regression). A brief description of the mixture modelling approach is presented in the Appendix.

With the hierarchical mixture regression model, a generalized linear mixed model (GLMM) was used to adjust for inter-community variations (via multilevel modelling) [28], where the community is represented in terms of postal area codes of participating mothers. The impacts of communities on the proportion of large for gestational age births are evaluated based on the "predicted" random effects [29]. A positive random (community) effect indicates an increased proportion of large for gestational age births in a community; see the Appendix.

For the mixture regression modelling presented in the Appendix, we first estimated the unknown parameters in the component densities with the adjustment for gestational age and baby gender. Based on the fixed estimated parameters in the component densities, risk factors were then included into the logistic regression function in steps, where each step corresponds to a single category of risk variables detailed in the study design above. Interactions between variables were considered at each step. For each category of risk variables, we performed the analysis included only individuals for which all variables in the category were present. Variables that were significant at 10% level (two-sided) within each category were entered into the final model for the determination of risk factors on the proportion of large for gestational age births. Obstetric complications of large for gestational age births were then determined by including the complication variables into the final estimated hierarchical mixture regression model via logistic regression.

The proportion of large for gestational age births in each community was calculated by averaging the estimated posterior probability of large for gestational age for all individuals in that community; see the Appendix. The estimated proportion of large for gestational age births was then compared to the unadjusted proportion of large for gestational age births, which was the estimated proportion of large for gestational age births in all regions without adjusting for the risk factors. Communities with more than ten participants and the proportion of large for gestational age births being higher than the unadjusted proportion of large for gestational age births were identified. These communities were associated with a higher than average proportion of large for gestational age births. Their characteristics in twelve pre-determined community profiles were explored based on the 2006 Australian Census of Population and Housing Community Profile data and the matching digital boundary base maps in generic Geographic Information System format [30].

## Results

### Sample characteristics

During the first three recruitment phases of the study (November 2006 to August 2008), the total number of mothers approached was 3321, of whom 1553 women (46.8%) agreed to participate and 1565 babies have been registered with the study (including twelve sets of twins).

The baseline characteristics of the recruited cohort are displayed in Table 1. The corresponding details of all births in the study region during 2006 are also presented to allow comparisons between cohort participants and the general population. The birth cohort sample did not differ significantly from the general population for maternal age or infant gender (Table 1). However, the percentage of infants with low birth weight (<2500 g) was approximately half that of babies born in the general population, due to the prospective mothers being recruited in the study towards the end of the third trimester. For the same reason, our sample did not include any infants born before 28 weeks gestation, and had a smaller proportion of infants born between 28 and 36 weeks gestation. In addition, the percentage of twins was approximately half of that in the general population, and our sample had a very small proportion of stillbirths. As the low birth weight and low gestational age groups in our sample are not good representatives of the population in general, the group of low birth weights (39, 2.5%), gestational age less than 37 weeks, and twin pregnancies are excluded from the analysis. There are a total of 1440 singleton babies with complete information on birth weight, gender, and maternal gestational age for the analysis.

**Table 1:** Baseline characteristics of the cohort and comparisons with all births in the study region

Characteristics	**Frequency (percentage**^**a**^**)**		**P-value**^**c**^
		
	Birth cohort sample (Years 2006 to 2008) n = 1565	**Deliveries in region**^**b**^ (Year 2006) n = 8608	
Gender of infant			
Male	764 (49.9%)	4462 (51.8%)	0.079
Female	782 (50.1%)	4145 (48.2%)	
Missing data	19	0	
Maternal age			
<20 years	87 (5.7%)	512 (5.9%)	0.239
20-24 years	325 (21.1%)	1608 (18.7%)	
25-29 years	424 (27.6%)	2388 (27.7%)	
30-34 years	427 (27.8%)	2515 (29.2%)	
≥35 years	274 (17.8%)	1584 (18.4%)	
Missing data	28	0	
Birth weight			
<2500 g	39 (2.5%)	450 (5.5%)	<0.0005
2500-3999 g	1266 (81.7%)	7073 (82.2%)	
≥4000 g	244 (15.8%)	1060 (12.3%)	
Missing data	16	2	
Gestational age at birth			
<28 weeks	0 (0.0%)	59 (0.7%)	<0.0005
28-36 weeks	38 (2.4%)	536 (6.2%)	
37-41 weeks	1505 (97.2%)	7963 (92.5%)	
≥42 weeks	6 (0.4%)	45 (0.5%)	
Missing data	16	4	
Plurality			
Singleton	1532 (98.5%)	8388 (97.4%)	0.016
Multiple	24 (1.5%)	220 (2.6%)	
Missing data	9	0	
Outcome			
Live birth	1554 (99.9%)	8547 (99.3%)	0.007
Stillbirth	2 (0.1%)	61 (0.7%)	
Missing data	9	0	

^a^Percentages are calculated based on the available (non-missing) data.

^b^Data for the study region (Logan, Gold Coast, Beaudesert, Tweed) are provided by Queensland Health and New South Wales Health.

^c^Chi-square test for comparing proportions between birth cohort sample and the general population.

### Birth weight

The adjusted mean birth weights at gestational age of 40 weeks for the first subgroup (corresponding to a group of infants of normal birth weight) are 3619 g (95% CI = 3580-3659) for males and 3488 g (95% CI = 3451-3524) for females. For the second subgroup (corresponding to a group of large for gestational age newborns), they are 4394 g (95% CI = 4231-4556) and 4249 g (95% CI = 4039-4458) for males and females, respectively. For comparison, we quote the 90^th ^and 95^th ^percentiles of Australian national birth weights at gestational age of 40 weeks from 1991 to 1994, which are 4170 g and 4340 g, respectively, for singleton males, and 4000 g and 4170 g, respectively, for singleton females [31].

### Proximal risk factors

The adjusted odds ratios of large for gestational age births for each category of risk factors are provided in Table 2. Several demographic (Pre-pregnancy obesity; Previous pregnancy; Marital status), socio-economic (Education level), and behavioural (Maternal smoking) factors have impact on risk of large for gestational age births. These five risk factors were entered into the final mixture model. The final results of determinants for risk of large for gestational age births are presented in Table 3.

**Table 2:** Categories of risk variables and adjusted odd ratios for large for gestational age (n = 1440)

Variable category	**Frequency (percentage**^**a**^**) or Mean (SD)**	Adjusted odd ratios (90% CI)
Demographics:		
Maternal age	28.90 (5.83)	0.96 (0.92, 1.01)
Pre-pregnancy (BMI) obesity^b^	211 (16.1%)	2.73* (1.64, 4.55)
Born in Australia	1039 (72.2%)	1.55 (0.85, 2.82)
Previous pregnancy	845 (58.7%)	2.26* (1.32, 3.88)
Married	732 (51.1%)	2.33* (1.36, 3.99)
Maternal work status (employed)	680 (47.6%)	1.22 (0.75, 1.97)
Missing data^c^	144 (10.0%)	

Socio-economics:		
House owned	619 (43.3%)	1.23 (0.74, 2.02)
Mother (education level)		
Not complete high school	281 (19.6%)	1.12 (0.63, 1.98)
Complete high school/TAFE (Reference)	883 (61.5%)	Reference
University degree	271 (18.9%)	0.45* (0.20, 1.00)
Household income		
Low (<$19,999)	81 (6.5%)	0.27 (0.05, 1.55)
Middle ($20,000-$80,000, Ref.)	835 (67.5%)	Reference
High (>$80,000)	322 (26.0%)	0.99 (0.54, 1.80)
Missing data	209 (14.5%)	

Psychological/Behavioural:		
No smoking during pregnancy	1100 (76.9%)	5.20* (2.12, 12.8)
Frequency of alcohol (at least weekly)	122 (8.5%)	0.72 (0.28, 1.90)
Vitamin supplements intake^d^	1085 (75.3%)	0.96 (0.58, 1.58)
Maternal mental health (very high risk^e^)	82 (5.8%)	0.17 (0.01, 5.13)
Missing data	51 (3.5%)	

Social network/Neighbourhood:		
Neighbours friendly or very friendly	975 (68.3%)	0.89 (0.52, 1.51)
Satisfied/very satisfied with community	1245 (86.8%)	1.08 (0.44, 2.67)
Community felt like home (agree)	1045 (73.3%)	1.17 (0.61, 2.23)
Get help when need it (agree)	1021 (71.3%)	0.93 (0.53, 1.63)
Get services need (agree)	1114 (78.3%)	0.96 (0.51, 1.82)
Feel safe (agree)	1128 (79.3%)	1.69 (0.75, 3.83)
Active in community (agree)	306 (21.6%)	0.93 (0.54, 1.62)
Moved home in past 1 year	632 (44.3%)	0.65 (0.40, 1.08)
Missing data	46 (3.2%)	

^a^Percentages are calculated based on the available (non-missing) data.

^b^BMI= (weight in kg)/(square of height in meter) ≥30.

^c^Number of individuals with incomplete data within each category of risk variables

^d^Any of supplements (Iron, Zinc, Calcium, Folic acid, Multi-vitamin, Vitamin C & E).

^e^Very high risk based on Kessler scale [45] (Kessler 6 scale >12)

**Table 3:** Determinants of risk and obstetric complications of large for gestational age

Variable	Coefficient	Adjusted odd ratios (95% CI)
**Determinants of risk of large for gestational age - **Demographics, Socio-economics, Psychological and Behavioural risk factors (n = 1294):		
Pre-pregnancy (BMI) obesity	1.006	2.73* (1.49, 5.01)
Previous pregnancy	0.707	2.03* (1.08, 3.81)
Married	0.614	1.85* (1.00, 3.42)
Mother education (university degree^a^)	-0.897	0.41 (0.16, 1.02)
No smoking during pregnancy	1.427	4.17* (1.43, 12.1)

**Obstetric complications of large for gestational age - **Birth procedures (n = 1235):		
Onset of labour		
Spontaneous (Reference)	Reference	
Induced	0.496	1.64 (0.85, 3.18)
Planned Caesarean section	-0.001	1.00 (0.42, 2.36)
Presentation (vertex)	-0.356	0.70 (0.46, 1.06)
Foetal distress	0.502	1.65 (0.52, 5.30)
Mode of delivery (Caesarean section or instrumental procedure)	0.683	1.98* (1.10, 3.55)

**Obstetric complications of large for gestational age - **Neonatal factors (n = 1282):		
APGAR score (5 minutes)	0.026	1.03 (0.97, 1.09)
Congenital anomaly	0.036	1.04 (0.31, 3.42)
Resuscitation procedures required	0.925	2.52* (1.33, 4.79)
Intensive or special care nursery	1.324	3.76* (1.89, 7.49)
Baby hospital length of stay >1 week	0.595	1.81 (0.52, 6.37)

^a^Relative to "no university degree" (not complete high school or complete high school/TAFE)

*Significant results at the 5% level.

There is an increased likelihood to have a large for gestational age baby (adjusted OR = 2.73, 95% CI = 1.49-5.01) for mothers who are categorized as obese during pre-pregnancy based on maternal pre-pregnancy BMI (Table 3). The likelihood of having a large for gestational age baby is also increased for mothers who have had a previous pregnancy (adjusted OR = 2.03, 95% CI = 1.08-3.81) and mothers who are married (adjusted OR = 1.85, 95% CI = 1.00-3.42). For mothers who did not smoke during pregnancy, there was an increased likelihood for giving birth to a large for gestational age baby (adjusted OR = 4.17, 95% CI = 1.43-12.1). The likelihood of having a large for gestational age baby is, however, decreased for mothers who have higher education level, though this result was only marginally significant at the 10% level. The assessment of subsequent obstetric complications of large for gestational age births is presented in Table 3. It was found that delivery of large for gestational age baby increases the chance of requiring caesarean section or instrumental procedure (adjusted OR = 1.98, 95% CI = 1.10-3.55). Also, newborns who are large for gestational age have a significantly higher likelihood of needing resuscitation procedures (adjusted OR = 2.52, 95% CI = 1.33-4.79) and admission to an intensive or special care nursery (adjusted OR = 3.76, 95% CI = 1.89-7.49).

### Community effect

**Figure 1:**
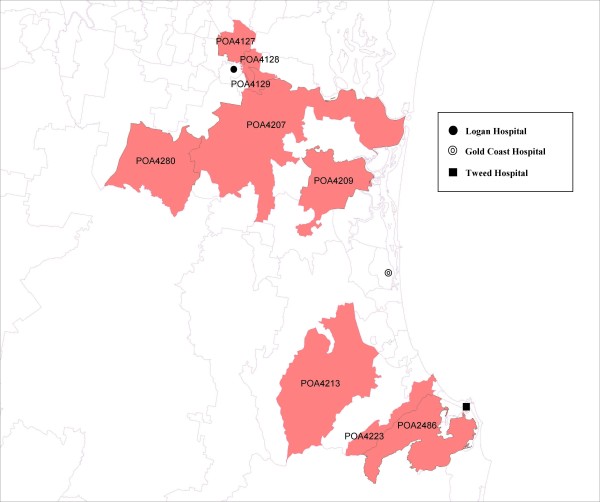
**Nine communities, designated by postal area (POA) codes, with a higher proportion of large for gestational age births**.

The unadjusted estimated proportion of large for gestational age births was 10.3%. We identified nine communities (postal code areas of participating mothers) that are associated with a higher proportion of large for gestational age births. These nine communities (Figure 1) have a higher proportion of mothers who possess some of those identified risk factors for large for gestational age babies. Of substantial importance is the finding that three of the communities (postal areas 2486, 4127, and 4280) have a large positive community effect (adjusted ORs are ranged from 1.7 to 2.2), that accounts for unknown adverse effects from the community other than those identified risk factors. The twelve pre-determined characteristics of the nine communities are presented in Table 4. Comparing to other communities, postal areas 4128 and 4129 have higher family incomes, higher labour force participations, but lower percentages of migrants moved in the community within a year. On the other hand, postal areas 4207, 4209, and 4213 have a lower population density but a higher proportion of females doing unpaid domestic work. The postal areas 4223 and 4280 have lower population densities and lower proportions of persons speaking other languages at home. They also have lower proportions of migrants moved in the community within a year. The postal area 2486 is unique; it has a higher percentage of indigenous persons but lower labour force participation.

**Table 4:** Characteristics of the communities with a high proportion of large for gestational age births

Characteristics	Postal area^a^									Others^b^ (n = 25)	P-value^c^
			
	2486	4127	4128	4129	4207	4209	4213	4223	4280		
Population density (person/km^2^)	242 (11)	727 (16)	1357 (23)	659 (15)	**125** **(6)**	**196** **(9)**	**109** **(5)**	**318** **(12)**	**92** **(3)**	1109 (21)	0.026
Indigenous persons	**3.1%** **(31)**	1.6% (21)	0.7% (4)	1.6% (20)	2.6% (28)	1.8% (22)	1.1% (12)	1.6% (19)	1.8% (23)	1.3% (15)	0.391
Other language spoken (home)	3.2% (3)	11% (25)	7.9% (19)	6.5% (16)	5.8% (11)	5.5% (10)	6.1% (12)	**3.4%** **(4)**	**3.6%** **(5)**	9.3% (21)	0.042
Family income ($/week)	921 (3)	1212 (27)	**1412** **(34)**	**1229** **(29)**	1107 (15)	1210 (26)	1181 (24)	1110 (16)	1198 (25)	1095 (13.5)	0.110
Household size (person)	2.4 (9.5)	2.7 (17)	3.0 (29)	2.9 (25)	2.7 (17)	3.0 (29)	3.0 (29)	2.5 (11)	3.2 (34)	2.7 (17)	0.105
Married persons	54% (28)	48% (15)	55% (29)	52% (23)	48% (14)	50% (18)	56% (31)	50% (21)	58% (32)	47% (13)	0.039
Volunteer	15% (27)	15% (28)	16% (29)	14% (18)	14% (23)	13% (11)	18% (33)	**15%** **(26)**	**15%** **(24)**	13% (14)	0.017
Unpaid domestic work (> 5 hours - females)	73% (28)	69% (20)	71% (25)	72% (26)	**71%** **(24)**	**76%** **(33)**	**71%** **(23)**	64% (13)	77% (34)	65% (14)	0.008
Age 20-39 ever born	66% (27)	55% (13)	58% (17)	59% (18)	66% (28)	65% (26)	63% (23)	57% (16)	73% (34)	55% (14)	0.086
One-parent family with children < 15	9.4% (17)	11% (26)	7.5% (5)	11% (24)	12% (27)	12% (29)	9.3% (15)	9.3% (16)	8.0% (6)	9.6% (18)	0.785
Labour force participation	**48%** **(2)**	68% (28)	**74%** **(34)**	**70%** **(31)**	62% (17)	72% (33)	67% (25)	59% (12)	67% (26)	61% (15)	0.051
Migrant lived at different address 1 year ago	**16%** **(3)**	17% (11)	**16%** **(6)**	**17%** **(8)**	17% (12)	38% (34)	17% (9)	**16%** **(5)**	**15%** **(1)**	20% (21)	0.008

^a^Values in parentheses are the rankings am ong the total of 34 communities.

^b^Median values (ranks) are presented among the 25 other communities.

^c^Mann-Whitney test for comparing medians between the nine communities and the others.

## Discussion

In this study, two sub-populations of infants were identified, with the first subgroup corresponding to a group of infants of normal birth weight and the second subgroup corresponding to a group of infants with large birth weight adjusted for gestational age and baby gender. We identified several risk factors that significantly increase the chance of having a large for gestational age baby. The findings improve the evidence-base regarding determinants and obstetric complications involving large for gestational age births. High maternal pre-pregnancy BMI has been shown to be related to high birth weight [2,3,32]. The association with parity, maternal age and region of birth was recently shown [16]. Our research findings indicate the adverse effect of maternal smoking on birth weights [3] and support previously reported results that reduced smoking prevalence among pregnant women partly explains the temporal increase in proportion of large for gestational age births [2,3]. Similar findings on the association between maternal smoking and low birth weight are demonstrated in the recent cohort studies conducted in Australia [33] and the UK [34]. Our results also confirm that the delivery of a large for gestational age infant is associated with an increased risk of obstetric complications such as caesarean delivery or instrumental delivery for mothers and the needs of resuscitation procedure or intensive/special care nursery for infants [35,36].

The identification of risk factors that are associated with large for gestational age infants has important public health implications. In the short-term, it is essential to target those pregnancies that have a high risk of having a large for gestational age infant and concomitant increased likelihood of obstetric complications. The large for gestational age infants will also have a higher risk of complications in the immediate post-delivery period. Hence they will require more intensive monitoring in the newborn nursery or neonatal intensive care unit [9]. There has been evidence that large for gestational age infants may have long-term health issues in addition to the short-term health complications mentioned above. These include an increased risk of suffering chronic diseases later in life such as diabetes, hypertension, and asthma.

We have identified nine communities that have a higher proportion of large for gestational age births. These communities have certain distinguishable characteristics from other communities in the study. They tend to have a higher proportion of females doing unpaid domestic work (except postal area 4223) but a lower proportion of migrants moving in the communities (except postal area 4209). Of substantial importance is the finding that three of the communities had a large positive community effect even after controlling for identified risk factors. While these findings will have the potential to pinpoint where improvements can be made within the community to reduce the impact of high birth weight on chronic health conditions and development in children, further validation on the findings are required when more data become available. The fourth-recruitment phase of the Environments for Healthy Living study has been completed and subsequent recruitment is scheduled for future years. This new birth cohort will help targeting interventions to reduce the escalating burden resulting from high birth-weight in Australia. It will also enhance the power to explore further the ecological determinants of large birth-weight and confirm research findings in other populations worldwide.

### Strengths and limitations

In this study, the mixture model assumed that the observed birth weights came from a population that consisted of two components corresponding to the appropriate for gestational age and large for gestational age subgroups. Thus we circumvented a major limitation in previous research in that the mixture modelling approach requires no prehoc threshold [37] to define large for gestational age infants. In contrast to the logistic regression approach that works on dichotomous outcomes of large for gestational age, the mixture modelling method attempts to model directly the birth weights, which are more informative relative to dichotomized outcomes for examining effects of risk factors. Another limitation of the logistic regression approach is that a pre-defined cut-off point for large for gestational age offers only a 'hard' classification of infants to large for gestational age and non-large for gestational age subgroups. This means that the estimated effects of the risk factors will be biased when there are substantially overlapping subgroups. The mixture modelling approach, on the other hand, offers a probabilistic classification of infants in the estimation of unknown parameters, and hence will provide less biased estimation of effect sizes [38].

Given there was complete follow up of subjects between the antenatal ascertainment of explanatory variables and the birth weight and obstetric outcomes the internal validity of the project is strong. The external validity of the results may be compromised by the sample recruitment method that did not engage women who are at risk for delivering babies with low birth weight. Similarly, women using private maternity services (normally those from higher socioeconomic backgrounds) and those with high risk pregnancies referred to specialist care are not captured in the sample. These shortcomings have been addressed in the analysis as described in the methods section by eliminating the group of low birth weights or gestational age smaller than 37 weeks.

In the analysis, we did not include maternal morbidities such as diabetes and hypertension as this information was not available for approximately 30% of the cohort due to differences in hospital perinatal data collection. As the national prevalence of these maternal co-morbidities are generally quite low (such as, 4.6% for gestational diabetes [39]), the number of women in our study sample who would have been affected would have been quite small. The association of diabetes and large birth weight has been demonstrated in previous cohort studies [9,10]. Moreover, it has been shown that weight gain during pregnancy is also related to large birth weight [40,41]. As this information was not available for approximately 55% of the cohort, it was not possible to perform multivariate analysis of large for gestational age births and potential risk factors with the inclusion of the weight gain during pregnancy without inducing serious bias in the estimation of adjusted odd ratios.

## Conclusions

Pre pregnancy obesity is the principal modifiable risk factor for large for gestational age births. Large for gestational age is an important risk factor for the subsequent obstetric complications. The findings from this new cohort study in Australia improve the evidence-base on which to base preventive interventions to reduce the impact of high birth weight on maternal and child health, and confirm research findings in other populations worldwide.

### Further study

With the Environments for Healthy Living study, follow-up routinely occurs when each child reaches 1 and 3 years of age. Participating mothers are mailed a questionnaire eliciting details on eco-epidemiological exposures and infant health and developmental outcomes including chronic markers for asthma, cardiovascular disease, and diabetes. In this research, 104 out of 1440 infants are classified as large for gestational age using the mixture modelling approach. Further study will focus on the comparisons of health and developmental issues between this group of children and the other who are not born large for gestational age. This research will improve the evidence base for the long-term health consequences of large for gestational age infants. Future data linkage with Medicare and related health data will also allow identification of health outcomes in this cohort. The longitudinal nature of the Environments for Healthy Living study has the advantage of being able to identify health and development issues in children in their early part of the life course, implement interventions that may minimize obesity problems among children and measure the long-term chronic disease problems in this group. Further research will compare maternal post-pregnancy weight retention between these two groups, as there is evidence that women retain their weight post-pregnancy have an elevated risk of entering their next pregnancy either overweight or obese [16,42].

## Competing interests

The authors declare that they have no competing interests.

## Authors' contributions

SN originated the research, led the design and implementation of mixture modelling, conducted the statistical analyses, and led the writing of the article. AO advised on the obesity, obstetrics, and gynecology aspects of the study. AS, CC, JS, and RM originated the Environments for Healthy Living study and supervised all aspects of its implementation. All authors assisted with conceptualizing ideas, interpreted research findings, and contributed to the writing of the article. All authors read and approved the final manuscript.

## Appendix: Hierarchical mixture regression modelling

Let *y_ij _*denote the baby birth-weight of the *j*th individual living in the *i*th community. With the mixture framework, the observed birth weights are assumed to have come from a mixture of two groups (corresponding to the normal and large for gestational age groups) in some unknown proportions that sum to one [38]. A two-component hierarchical-mixture-regression model is represented by

$$f(y_{ij},x_{ij},x_{ij}^{'})=(1-\pi (x_{ij}^{'}))\phi_{1}(y_{ij},x_{ij})+\pi (x_{ij}^{'})\phi_{2}(y_{ij},x_{ij}),$$

where $\pi (x_{ij}^{'})$ denotes the probability of the *j*th individual belonging to the second component corresponding to the subgroup of large for gestational age and *ϕ_k_*(*y_ij_*, *x_ij_*) is the *k*th component-density function (*k *= 1, 2). The proportion *π *is specified as a logistic function of $x_{ij}^{'}$,

$$\pi (x_{ij}^{'})=\frac{\text{exp}(a+b^{T}x_{ij}^{'}+U_{i})}{1+\text{exp}(a+b^{T}x_{ij}^{'}+U_{i})},$$

where $x_{ij}^{'}$ is a vector of risk factors or complication variables associated with *y_ij _*and *U_i _*represents the unobservable random effect due to the *i*th community affecting on the proportion *π*. These (random) community effects are taken to be normally distributed with zero mean and variance λ [28]. A positive random effect *U_i _*indicates an increased proportion of large for gestational age births in a community. It is further assumed that the component densities are normally distributed with a common variance σ^2 ^[38], and the mean is expressed in terms of *x_ij _*corresponding to the variables of gestational age and baby gender. The posterior probability of a large for gestational age birth for the *j*th individual in the *i*th community is given by $\tau_{ij}=\pi (x_{ij}^{'})\phi_{2}(y_{ij},x_{ij})/f(y_{ij},x_{ij},x_{ij}^{'})$. Let *n_i _*be the number of participants in the *i*th community, the proportion of large for gestational age births in the *i*th community is given by the averaged posterior probability $\sum_{j}\tau_{ij}/n_{i}$. The unknown parameters are estimated using the GLMM approach [28] via the expectation-maximization (EM) algorithm [43]. In particular, the posterior probabilities are computed based on current estimates of model parameters; they lie between zero and one and offer a probabilistic classification of infants with respect to the normal and large for gestational age groups; see, for example, [44]. Model selection and goodness-of-fit can be assessed based on information criteria or the likelihood-ratio test statistic [28]. A Fortran program for the estimation of model parameters is available on request from the first author.

## Pre-publication history

The pre-publication history for this paper can be accessed here:

http://www.biomedcentral.com/1471-2458/10/460/prepub
